# Philadelphia County Medical Society

**Published:** 1890-12

**Authors:** 


### PHILADELPHIA COUNTY MEDICAL SOCIETY.

STATED Meeting, September 24, 1890.

The Vice-President, JOHN B. ROBERTS, M. D., in the chair.

Dr. CHARLES B. PENROSE read a paper on

### THE TREATMENT OF HEMORRHOIDS BY EXCISION.

My object in presenting this paper is to urge the more general use of Whitehead's operation of excision in the treatment of certain cases of hemorrhoids.

In 1887, Mr. Whitehead, of Manchester, reported[1] three hundred consecutive cases of hemorrhoids which had been successfully treated by the method of excision and suture. His operation is performed in the following manner:

1. The patient is placed on a table in the lithotomy position, with the hips well elevated.

1. *British Medical Journal*, February 6, 1887.

2. The anal sphincters are then thoroughly paralyzed by digital stretching.

3. The mucous membrane of the rectum is divided at its junction with the skin around the entire circumference of the bowel.

4. The mucous membrane, with the attached hemorrhoids, is dissected from the submucous tissue, and the cuff or cylinder thus formed is dragged below the skin margin.

5. The mucous membrane above the hemorrhoids is then divided transversely, thus removing the pile-bearing area, and the operation is completed by suturing the upper margin of the severed membrane to the free margin of the skin.

The advantages claimed by Whitehead for this method of treatment are based on pathological and on surgical reasons. He considers that the internal hemorrhoids, which are generally regarded as localized distinct tumors, amenable to individual treatment, are, as a matter of fact, component parts of a diseased condition of the entire plexus of veins surrounding the lower rectum, each venous radicle being similarly, if not equally, affected by an initial cause, constitutional or mechanical.

The operation of excision is the only one which removes this whole diseased area. It is, therefore, demanded for this pathological reason. It is, in addition, surgically more perfect than any other method of treatment, because it provides for the readjustment of healthy tissues, with the object of securing primary union and rapid convalescence. It does not leave the sluggish ulcer of the cautery, nor is it attended with the pain and slow convalescence of the ligature.

My experience with this operation is limited to ten selected cases. Only those cases were selected in which there existed a complete circle of hemorrhoidal tumors surrounding the lower margin of the rectum, since for such cases Whitehead's treatment of excision seems to be most particularly adapted.

The details of the operation are simple and easy to execute. In dividing the mucous membrane from the skin, it is best to begin at the posterior margin of the anus in order to prevent the blood from obscuring the field of operation. No skin should be sacrificed, even though there appear to be redundant tags around the margin of the anus.. The skin always retracts somewhat and the tags shrivel and disappear before firm union has taken place. Failure to observe this rule may result in subsequent serious trouble. Kelsey[1] reports the case of a woman who had been subjected to a so-called Whitehead operation, and who presented herself to him with a complete circle of excoriated mucous membrane, extending for one inch outside the anus. It is probable that in this case the operator had sacrificed too much skin.

On the other hand, the upper section of the mucous membrane should be made in the same horizontal plane throughout, in order to prevent subsequent ecropion ani.

The dissection of the mucous membrane from the underlying tissue is exceedingly easy, except in some cases of old — or long-standing — piles. The attachment of the sub-mucous tissue is very loose, and separation can be effected with the finger or with the handle of the scalpel. It is not always possible to dissect the piles completely from the underlying structures, as they may involve not only the mucous but the sub-mucous tissues, and in such cases it is necessary to cut partly through the piles until the healthy mucous membrane above is reached. Repeated attacks of inflammation, of course, render closer the adhesion of the pile area to the underlying structures. In one of my own cases, where the piles had existed for forty years, and had frequently been inflamed, the adhesions to the two sphincters were so close that a few muscular fibres were cut away during the removal.

The amount of blood lost during the operation is surprisingly small. Whitehead states that he has often operated on severe cases and not found it necessary to twist a single vessel. In five of my cases no hemostasis was necessary. Bleeding is avoided by adhering closely to the mucous membrane in the dissection, as the larger arterioles lie beneath the submucous tissue. The arterial bleeding occurs in those cases of old piles which have been subjected to previous operation or to attacks of inflammation, and in which dilatation of the rectal and anal arteries has taken place secondary to dilatation of the hemorrhoidal veins. The bleeding from the upper divided edge of the mucous membrane can be reduced to a minimum by following Whitehead's method of inserting the sutures, as each portion is divided, or by adopting Marcy's plan of introducing a circle of shoemaker stitches of catgut around the mucous membrane above the piles before cutting the mass away.

Whitehead's advice is in all cases to remove the complete cylinder of mucous membrane, whether or not the whole of this area appears to be diseased. He gives this advice for the reason, which

1. *New York Medical Journal*, October 5, 1889.

I have already stated, that he considers the individual piles as but part of a general pathological condition, involving all the lower hemorrhoidal veins of the rectum.

Whether we accept this pathological view or not, it is best to follow this plan, and to make a complete circular division of the mucous membrane, as by this method the best surgical results are obtained, and ectropion ani prevented. I have seen a case in which only one-half of the circumference of the mucous membrane of the rectum was removed, and a few hours after the operation an edematous swelling formed in the other half, which has now resulted in a hemorrhoidal tumor almost as annoying as the one for which the operation was performed.

In attaching the mucous membrane to the skin, Whitehead uses the interrupted silk suture. He never removes the sutures, but allows them to ulcerate through — a process which is very easily accomplished. In my own cases I have used the continuous catgut suture.

The treatment of these cases after operation is very simple. It is rarely necessary to use opium or the catheter. An opium and belladonna suppository introduced immediately after the operation, is in most cases all that is required. The bowels can be moved in from twenty-four hours to four days, and with very little pain. Absence of pain after Whitehead's operation is due to the thorough paralysis of the sphincters, and to the fact that no source of irritation is left beyond that of a clean linear incision, united without tension and without strangulation of tissue.

A glance at the histories of my own cases shows that they were all cases of aggravated hemorrhoids, in which the piles covered the whole circumference of the lower part of the rectum. In all the cases the disease had existed for many years, and two had been subjected to previous operation by the ligature.

In only one case was there anything like free bleeding during the operation.

In all the cases a suppository of one-half grain of extract of opium and one-half grain of extract of belladonna was introduced immediately after the operation, and this was all the opium required, except in three cases, in which one-sixth grain of morphine was subsequently administered.

The catheter was used in only three cases, and in these for a period not longer than twenty-four hours. The length of time that the case is confined to bed depends to a great degree upon the social standing and the disposition of the patient. In my cases it varied from two to ten days. Every case should be able to sit up in four or five days, and to resume work in ten days or two weeks.

The bowels were opened without pain in from twenty-four hours to four days after the operation.

No complications of any kind followed these operations. Union takes place quickly, and generally one dressing, taken off when the bowels are moved, is all that is necessary. In no case was there incontinence from paralysis of the sphincters, or any tendency to stricture from contraction of the scar.

Since the publication of Whitehead's paper, his method of operating has been tested by many surgeons. The operation cannot be criticised on surgical grounds, as it is certainly the most perfect plan of treatment, surgically speaking, which has been proposed.

The immediate removal of the tumors, the coaptation of healthy tissues, and primary union, are substituted for slow strangulation by the ligature, or removal by the cautery and healing by granulation.

The applicability, or the necessity, of this operation in all cases of hemorrhoids is, however, open to criticism. If we accept Whitehead's views in regard to the pathology of piles, and believe that the whole venous plexus surrounding the anus and the lower end of the rectum, is in a pathological condition in every case of hemorrhoids, even though there may be present only one or two isolated tumors, then, of course, the complete removal of this area is indicated.

But that this view is not true, is proved by the thousands of cases which have been permanently cured by the ligature and the clamp. The method, however, is indicated in all cases of aggravated hemorrhoids where the vascular tumors cover the whole or the greater part of the circumference of the bowel. In such cases the operation presents no great difficulties. Statistics show that it is at least as safe as operation by the ligature or the clamp, and it is certainly followed by a more rapid convalescence and much less pain and discomfort.

DISCUSSION.

Dr. W. D. Green: I have had the pleasure of witnessing only a small number of Whitehead's operations, but I fully agree, and I think that those who have tried the operation will fully agree with Dr. Penrose, that the method of excising through the whole circumference of the bowel, the pile-bearing mucous membrane, and drawing down upon the upper segment and then attaching this to the lower segment without including the skin, has the advantages, first, of removing all possibility of return of the trouble ; and, secondly, as Dr. Penrose has stated, in making a clear, clean, linear incision around the circumference of the bowel. Nearly all of us have seen the immense amount of suffering which the older operations, by means of the clamp and ligatures, and even the cautery, have entailed. In the cases of the new operation, which I have seen, recovery has been rapid and complete. In one case, that of a woman well advanced in life, upon the day after operation, when I got to the house, I found her comfortably seated in a rocking-chair. The physician who had had the case in charge before the operation had given her freely of some medicine to open the bowels, and on the morning after the operation, without any pain, without any tenesmus, she had a large, well-formed motion. In the old method, in which for days the physician was called upon to administer opium, either by suppository or hypodermically, in large amounts, and in which the patient and the physician both looked forward with dread to the time — five, six, or ten days after the operation — when the bowels were to be opened, is by this method entirely obviated.

I have seen, in the few cases which I have watched, no pocketing or trouble about the line of incision. The two freshly cut surfaces unite very quickly — very much more so, it seems to me, than in mucous surfaces elsewhere. Even when the bowels were moved within twenty-four or thirty-six hours, I was surprised to find that there was no trouble.

It strikes me that the continued suture has advantages over the interrupted. Being introduced and made fast at one point, and then carried out and in around the circumference of the bowel, if catgut be used at the time when union usually occurs the suture is probably dissolved and passes away without any trouble ; or, if silk be used, by simply introducing the scissors and cutting close to the knot and giving an easy pull, the whole suture is removed without any pain or bleeding.

I must confess that the operation presents to me by far the best method of removing the pile-bearing membrane when it exists and involves one-half or more than one-half the membrane around the circumference of the bowel.

---

THE REMOVAL OF FRECKLES.—The *Pharmaceutical Record* quotes the following prescription for removing freckles :

| | | |
|---|---|---|
| ℞.— | Sulphocarbolate of zinc................ | 1 drachm. |
| | Glycerin ............................ | 2 ounces. |
| | Alcohol.............................. | 1 ounce. |
| | Orange-flower water.................. | 1½ ounce. |
| | Rose water, sufficient to make.......... | 8 ounces.—M. |

Apply twice daily.

